# Mobilization of Peripheral Blood Stem Cells and Changes in the Concentration of Plasma Factors Influencing their Movement in Patients with Panic Disorder

**DOI:** 10.1007/s12015-016-9700-6

**Published:** 2016-12-02

**Authors:** Marcin Jabłoński, Jolanta Kucharska Mazur, Maciej Tarnowski, Barbara Dołęgowska, Daniel Pędziwiatr, Ewa Kubiś, Marta Budkowska, Daria Sałata, Justyna Pełka Wysiecka, Arkadiusz Kazimierczak, Artur Reginia, Mariusz Z. Ratajczak, Jerzy Samochowiec

**Affiliations:** 1 Department of Psychiatry, Pomeranian University of Medicine, Broniewskiego 26, 71-460 Szczecin, Poland; 2 Department of Physiology, Pomeranian University of Medicine, Szczecin, Poland; 3 Department of Medical Analytics, Pomeranian University of Medicine, Szczecin, Poland; 4 Department of Vascular Surgery, Pomeranian University of Medicine, Szczecin, Poland; 5 Stem Cell Biology Program at the James Graham Brown Cancer Center, University of Louisville, Louisville, KY 40202 USA

**Keywords:** VSEL, Panic disorder, Sphingosine - 1 – phosphate, SDF-1, Complement components, Stem cells

## Abstract

In this paper we examined whether stem cells and factors responsible for their movement may serve as new biological markers of anxiety disorders. The study was carried out on a group of 30 patients diagnosed with panic disorder (examined before and after treatment), compared to 30 healthy individuals forming the control group. We examined the number of circulating HSCs (hematopoetic stem cells) (Lin−/CD45 +/CD34 +) and HSCs (Lin−/CD45 +/AC133 +), the number of circulating VSELs (very small embryonic-like stem cells) (Lin−/CD45−/CD34 +) and VSELs (Lin−/CD45−/AC133 +), as well as the concentration of complement components: C3a, C5a and C5b-9, SDF-1 (stromal derived factor) and S1P (sphingosine-1-phosphate). Significantly lower levels of HSCs (Lin−/CD45 +/AC133 +) have been demonstrated in the patient group compared to the control group both before and after treatment. The level of VSELs (Lin−/CD45−/CD133 +) was significantly lower in the patient group before treatment as compared to the patient group after treatment.

The levels of factors responsible for stem cell movement were significantly lower in the patient group compared to the control group before and after treatment. It was concluded that the study of stem cells and factors associated with their movement can be useful in the diagnostics of panic disorder, as well as differentiating between psychotic and anxiety disorders.

## Introduction

According to the current version of the International Statistical Classification of Diseases and Related Health Problems (ICD-10), panic disorder may be diagnosed in a situation of occurrence of a few severe anxiety attacks with concurrent autonomic symptoms within the period of one month. Panic attacks are unrelated to a real situation of threat, they appear unrelated to a known or predicted situation, between attacks one is relatively free from symptoms of anxiety [1]. In research by Grant et al., lifetime prevalence of panic disorder (PD) is 5.1 %, in the case of PD without agoraphobia 4.0 % and in panic disorder with agoraphobia 1.1 % [2]. The etiology of anxiety disorders is not clear, it is believed that both environmental and genetic factors are responsible for their occurrence. In their meta-analysis, Hettema et al. estimated the heritability of PD at 0.48 (95 % CI:. 41–54) ***‘***[3]. For both panic disorder and generalized anxiety disorder the MZ: DZ concordance ratio was more than 2:1 [4], Mendlewicz et al. showed a significantly higher risk of developing PD among relatives of patients with PD [5].

Linkage analyses showed several regions on chromosomes which may be related to PD (1q 2q (14), (15), 4q31-q34 (16, 17), 7 p (18, 19), 9q (17, 20), (21) 12q, 13q (17, 22), 14q (17, 23), 15q (15), and 22q (17, 24). Association studies on candidate genes that may be associated with PD investigate, among others, COMT genes, 2 TPH, MAOA, 5-HT, 5-HTT [6–13].

Reports on the importance of regenerative processes in the etiology of anxiety disorders are extremely scarce, and it is worth pointing out that increased levels of stem cells are found in the peripheral blood in different models of major illnesses or deficits related to a significant level of stress (myocardial infarction, burns, inflammatory bowel disease, stroke). Therefore, their level in the peripheral blood can have potentially diagnostic and predictive value in any of the disorders in which there appears an increased stress level [14–18].

Animal models of stress provide information on the reduction of endogenous neurogenesis in the dentate gyrus [19]. It is postulated that the process of neurogenesis is involved in the pathogenesis of depression [20].

In the study by Reif et al., it has been shown that weakening of the neurogenesis process may play a role in schizophrenia, but it does not seem to be important in affective disorders [21]. Kucharska et al. investigated the relationship between the first episode of psychosis and HSC and VSEL levels. They showed that in a group of people experiencing their first psychotic episode in comparison with the control group, the VSEL level is significantly higher [22]. The use of psychiatric drugs has an effect on the process of neurogenesis. It has been shown, for example, that the administration of fluoxetine in adult rats increases the proliferation of new neurons and reduces the impact of stress on cell proliferation [23, 24]. Kigawa et al. indicated a higher effectiveness of neural stem cell therapy, as well as sertraline and neural stem cell therapy in the treatment of drug-resistant depression in animal models, compared with animals treated only with sertraline **[** 25].

In our research we investigated the behavior of HSCs (hematopoetic stem cells) and VSELs (very small embryonic-like stem cells). HSCs are stem cells, isolated from the bone marrow or peripheral blood (1/10,000), which may give rise to 8–10 lines of blood cells. At the turn of the II-III trimester of pregnancy they colonize the bone marrow, thanks to the SDF-1 (stromal derived factor-1) produced by stromal cells and osteoblasts, which is a factor that plays a role in their retention in the bone marrow [26, 27]. Their mobilization from the bone marrow happens with the participation of G-CSF (granulocyte colony-stimulating factor). The cells isolated from the bone marrow have the phenotype CD 34 +/CD38- [28].

VSELs are pluripotent stem cells with the phenotype of CXCR4 +, AC133 +, CD34 +, SSEA-1 + (mouse), SSEA-4 + (human), AP +, c-Met+, LIF-R +, CD45-, Lin-, HLA-DR-, MHC I −, CD90-, CD29-, CD105-, we isolate them from the bone marrow, umbilical cord blood and peripheral blood [26]. They are found in the bone marrow, as a result of migration in the early stages of the ontogenetic development, and are deposited in the mature bodies, including the brain [29–32]. VSELs found in the peripheral blood could potentially serve as a repair system in case of damage, including damage to the CNS. It has been shown that they are mobilized to peripheral blood in a mouse model of stroke but also in the early phase of ischemic stroke in humans [16, 33]. There is also some hope to apply them in the repair processes of the retina [34].

In the body, stem cells are deposited in areas called niches, they move thanks to a variety of factors which include the components of the complement cascade, bioactive chemokines and lysophospholipids [16, 35, 36]. An important role in the regulation of those processes play: SDF-1 (stromal derived factor), S1P (sphingosine-1-phosphate) and components of the complement cascade. The interaction between SDF-1 and CXCR4 receptor is responsible for the maintenance of stem cell in the niches. Apart from the bone marrow, such niches are found for example in the brain in the periventricular zone of the lateral ventricles and the olfactory bulb, as well as the subgranular zone of the hippocampus dentate gyrus [37–39]. SDF-1 is involved in the development of the CNS, as well as in the ongoing processes of neuromodulation, neurogenesis and neuroprotection [40]. Receptors for S1P are present on oligodendrocytes, neurons and nerve progenitor cells [41–43]***.*** It takes part in neurogenesis, plays an important role in directing stem cells to the nerve tissue, stimulates the migration of progenitor cells to damaged areas in the spinal cord [44], and affects the growth and differentiation of oligodendrocytes [43]. Administration of migration inhibitors, the mediator of which is S1P is used in multiple sclerosis therapy [45].

Anaphylatoxins C3a, C5a and C5b-9 have a significant impact on the neuroglia and CNS neurons [46]. C3a component affects the colonization of the bone marrow niches by stem cells, stimulates chemotaxis as well as hematopoietic and progenitor cell retention in the bone marrow [47–49]. Lack of the final activation of the complement cascade in mice results in a significant defect within mobilization of HSCs [50]. Microglia is a mediator in the synthesis of C3 and C5 in the brain and the emergence of C3a and C5a that are vasoactive and chemotactic agents for neutrophils [51], while C5a has a neuroprotective effect on mature cells [52]. On the CNS neurons there are receptors for C5a, which respond to stimulation by C5a in a stressful situation in the form of ischemia [53]. Based on the above information and the fact that reports on the role of stem cells in mental disorders are sparse, we decided to see if the stress present in panic disorder significantly affects the mobilization of stem cells and the plasma concentration of factors responsible for their movement.

## Material and Methods

### Patient Group and Control Group

The study was approved by the Ethical Commission of the Pomeranian Medical University. All the individuals participating in the study provided their written consent. The investigated group consisted of 30 patients, 12 of whom were diagnosed with panic disorder with agoraphobia and the remaining 18 with panic disorder without agoraphobia according to ICD-10. The participants did not suffer from other concurrent diseases or mental disorders. The patients were compared to an ethnically, BMI and gender-matched control group of 30 healthy volunteers without any diet restrictions or psychiatric disorders, which were excluded through an examination by a specialist psychiatrist. As is well known, the occurrence of psychological disorders can be affected by events such as complications related to pregnancy or childbirth, or certain family issues so the groups were matched in this respect too (Table 1). All persons with a history of serious medical events during their lifetime (including glucose intolerance), organic brain injuries or drug/alcohol dependence were excluded from the study.

**Table 1:** Study groups: demographics and clinical features

	PG (*n* = 30)	CG (*n* = 30)	P
Mean age (years)	39,73 ± 9,4	34,13 ± 11.78	0,053
Gender	Male - 7 (23,3 %)	Male - 10 (33,33 %)	0,284
	Female – 23 (76,67 %)	Female - 20 (66,67 %)	
Education	Elementary – 0(0 %)	Elementary – 1 (3,33 %)	0,24
	Vocational school – 5 (17,86 %)	Vocational school – 1 (3,33 %)	
	Secondary −10 (35,71 %)	Secondary −13 (43,33 %)	
	Higher – 13 (46,43 %)	Higher – 15 (50,0 %)	
Pregnancy – any complications	Yes – 6 (20 %)	Yes – 3 (10 %)	0,27
	No – 24 (80 %)	No – 27 (90 %)	
Natural childbirth	Yes – 26 (86,67 %)	Yes – 27 (90 %)	0,68
	No – 4 (13.33 %)	No – 3 (10 %)	
Divorce of parents	Yes – 8(26,67 %)	Yes – 3 (10 %)	0,09
	No – 22 (73,33 %)	No - 27 (90 %)	
Family history of psychotic disorders	Yes – 3 (10 %)	Yes – 6 (20 %)	0,27
	No – 27 (90 %)	No – 24(80 %)	
Smoking subjects	Yes – 15(55,56 %)	Yes – 11 (36,67 %)	0,15
	No – 12 (44,44 %)	No – 19 (63,33 %)	
BMI	24,75 ± 4,8	24.61 ± 4.33	0,90
Mean duration of anxiety (days)	1564 ± 2297	-	
Mean duration of treatment(days)	24,47 ± 16,26		

The diagnosis of PD was conducted by means of a psychiatric examination, MINI [54] and according to ICD-10 criteria. Demographic data, family history and history of symptoms were assessed by a structured interview; we collected information from the patients and their family. In order to assess the severity of the anxiety disorder we applied the HAM-A [55], to evaluate depressive disorders we used the MADRS [56] (Table 2)

**Table 2:** Mean MADRS and HAM-A scores in patient group before and after treatment

	Mean score before treatment	Mean score after treatment	p
HAM-A	22,2 ± 9,08	4,03 ± 5,57	0,00001
MADRS	8867 ± 5,82	1,9 ± 4413	0,00004

.

### Research Procedures

Venous blood samples were collected in the morning, on an empty stomach. We collected blood twice in the patient group, the first time before they received medication, the second time after observed clinical improvement, defined as withdrawal of panic attacks and after the improvement in clinical scales (Hamilton, MADRS) to a normal level. Psychiatric assessment, psychometric evaluation, physical examination, and laboratory tests were performed on the same day. Pharmacological treatment was administered in accordance with Polish standards of treatment of anxiety disorders.

### Flow Cytometric Analysis

Peripheral blood (PB) samples were lysed twice using BD Pharm Lyse lysing buffer (BD Bioscience) at room temperature for 10 min and subsequently washed in phosphate-buffered saline (PBS) with 2 % fetal bovine serum (FBS; Sigma) to yield total nucleated cells (TNCs). TNCs were stained for hematopoietic lineage markers using the following fluorescein isothiocyanate (FITC)-conjugated antibodies (Abs) against human proteins: CD2 (clone RPA-2.10); CD3 (clone UCHT1); CD14 (clone M5E2); CD16 (clone 3G8); CD19 (clone HIB19); CD24 (clone ML5); CD56 (clone NCAM16.2); CD66b (clone G10F5); and CD235a (clone GA-R2) (all from BD Bioscience). The cells were simultaneously stained for the panleukocytic marker, CD45 phycoerythrin (PE)-conjugated Abs, (clone HI30; BD Biosciences) and one of the following antigens CD 34-allophycocyanin (APC)-conjugated Abs, (clone 581; BD Bioscience) or CD133 (CD133/1, APC-conjugated Abs; Miltenyi Biotec). Additionally following FITC-conjugated isotype controls: Mouse IgG1, (clone MOPC-21), Mouse IgG2a, (clone G155–178), Mouse IgG2b, (clone 27–35), PE-conjugated isotype control Mouse IgG1, (clone MOPC-21) and APC-conjugated isotype control Mouse IgG1, (clone MOPC-21) (all from BD Bioscience) were used. In addition, APC-conjugated isotype control Mouse IgG1 (clone IS5-21F5; Miltenyi Biotec) were used. Staining was performed in PBS with 2 % FBS on ice for 30 min. Cells were subsequently washed, resuspended, and analyzed using an NAVIOS Flow Cytometer (Beckman Coulter). At least 10^6^ events were acquired from each sample. The absolute numbers of VSEL and the absolute number of white blood cells were calculated (individually for each patient) per 1 ml PB based on the percent content of these cells as detected by flow cytometry. Kaluza software (Beckman Coulter) was used for analysis [16, 57].

### Determination of S1P by Reversed-Phase High-Performance Liquid Chromatography

Fresh blood samples were centrifuged (250 g; 10 min., 20 °C), and the plasma was frozen at −80 °C for further study. Plasma was defrosted at room temperature, the internal standard (D-erythro-sphingosine-1-phosphate, S1P C17, Avanti Polar Lipids), 1 M NaCl, methanol and 37 % HCl was added, and the samples were vortexed. Chloroform (2 ml) was added, and the samples were mixed in a test tube rotator. Samples were centrifuged, and the lower chloroform phase was transferred into a new tube. Following a re-extraction with another 2 ml of chloroform, the chloroform phases were combined and vacuum-dried in a SpeedVac for 45 min at 45 °C. The residue that remained after evaporation was reconstituted in methanol, incubated with OPA (o-phthaldialdehyde), methanol, mercaptoethanol and boric acid (pH 10,5) and centrifuged. The supernatant was analyzed using a Hewlett Packard Series 1200 chromatograph. Chromatographic data were processed by HP Chemstation software (Hewlett Packard, now Agilent). Reversed-phase HPLC was performed on a Cosmosil C18-ARII column (250 mm × 4 mm, 5 μm) (Phenomenex) with a C18-ARII cartridge (10 mm × 4,6 mm) packed with the same material. The column temperature was 25°C. An isocratic method was used where the mobile phase was composed by mixing K_2_HPO_4_ (pH 5,5) and methanol (15:85; *v*/v). The flow rate was 1 ml/min, and 50 μl samples were injected every 30 min. Detection was performed with a fluorescence detector at 340 nm excitation and 455 nm emission wavelengths. The quantification was based on peak areas with internal standard calibration [58–60].

#### Real-Time Quantitative Reverse Transcription PCR (qRT-PCR)

Total RNA was isolated from lysed blood with the RNeasy Kit (Qiagen). The RNA was reverse transcribed with FirstStrand cDNA synthesis kit and oligo-dT primers (Fermentas). Quantitative assessment of mRNA levels was performed by real-time RT-PCR on an ABI 7500Fast instrument with Power SYBR Green PCR Master Mix reagent. Real-time conditions were as follows: 95 °C (15 s), 40 cycles at 95 °C (15 s), and 60 °C (1 min). According to melting point analysis, only one PCR product was amplified under these conditions. The relative quantity of a target, normalized to the endogenous control β-2 microglobulin gene and relative to a calibrator, is expressed as 2-∆∆Ct (−fold difference), where Ct is the threshold cycle, ∆Ct = (Ct of target genes) – (Ct of endogenous control gene, β-2 microglobulin), and ∆∆Ct = (∆Ct of samples for target gene) – (∆Ct of calibrator for the target gene) [16, 61].

#### Determination of C5b-9, C3a And C5a Complement Components and SDF-1

C5b-9 (MAC), C3a and C5a complement components were determined by using the Human C5b-9 ELISA Kit, Human C3a ELISA Kit and Human C5a ELISA Kit (BD OptEIA), respectively.

SDF-1 was determined using a Human CXCL12/SDF-1 ELISA Kit (R&D Systems) [58, 62–64].

### Statistical Analysis

Distribution of continuous variables was examined with the Shapiro-Wilk test. Comparison of quantitative variables was performed with the Mann-Whitney-U test. Relative continuous variables were compared with the Wilcoxon test. Dichotomous variables comparison was performed with the use of the exact Fisher test. As the measure of statistical significance we used *p* < 0.05. All statistical analyses were performed using Statistica 10, by StatSoft, 2013.

## Discussion

In the course of our research, we looked for the answer to the question of whether anxiety disorders affect the mobilization of stem cells into the peripheral blood, and hence, whether stem cell concentration and associated humoral factors can become a new diagnostic tool for anxiety disorders. Our study showed a statistically significant decrease in the level of hematopoietic stem cells HSCs (Lin−/CD45 +/AC133 +) in the patient group as compared to the control group both before and after treatment (Table 3). Available publications do not include analyses concerning panic disorder and the related behavior of stem cells. Therefore, we investigated the impact of stress on stem cells. Wohleb et al. demonstrated that mice subjected to the influence of repeated stress exhibit anxiety disorder type behaviors associated with an increase in the number of monocytes, cytokines and chemokines involved in the recruitment of myeloid cells as well as an increase in the number of monocytes in particular brain structures. In contrast, mice with chemokine receptor 2 (CCR2) knockout or fractalkine receptor (CX3CR1) knockout do not exhibit such behavior and are not able to recruit macrophages to the brain [65].

**Table 3:** Comparison of stem cells concentrations and factors responsible for their movement in PG BT vs CG and comparison of the mean number of stem cells and factors responsible for their movement in PG AT vs CG.

	PG BT (*n* = 30)	CG (*n* = 30)	P	PG AT (*n* = 30)	CG (*n* = 30)	P
VSELs (Lin−/CD45−/CD34+) [No.of cells/μl]	0,155 ± 0,079	0,160 ± 0,103	0,82	0,158 ± 0,064	0,160 ± 0,103	0,93
VSELs (Lin−/CD45−/CD133+) [No. of cells/μl]	0,102 ± 0,048	0,116 ± 0,073	0,38	0,134 ± 0,074	0,116 ± 0,073	0,34
HSCs (Lin−/CD45+/CD34+) [No. of cells/μl]	1173 ± 0,522	1234 ± 0,692	0,7	1265 ± 0,62	1234 ± 0,692	0,85
HSCs (Lin−/CD45+/AC133+) [No.of cells/μl]	0,860 ± 0,43	1229 ± 0,736	0,02	0,897 ± 0,45	1229 ± 0,736	0,04
C3a [ng/ml]	469,56 ± 116,6	586,67 ± 136,2	0,00071	449,9 ± 86,13	586,67 ± 136,2	0,00002
C5a [ng/ml]	27 ± 13	54,11 ± 28,9	0,00001	26,98 ± 12,67	54,11 ± 28,9	0,00001
C5b-9 [ng/ml]	73,97 ± 77,29	235,95 ± 317,5	0,0087	61,43 ± 39,76	235,95 ± 317,5	0,0041
SDF-1 [pg/ml]	2101 ± 401	2828 ± 395	0,00001	2057,4 ± 422	2828 ± 395	0,00001
S1P [μg/ml]	2,05 ± 0,38	2,48 ± 0,13	0,00001	2,00 ± 0,32	2,48 ± 0,13	0,00001

In the study by Heidt et al. it has been shown that chronic stress increases the level of monocytes and neutrophils in humans, whereas the search for the source of leukocytosis in mice suggested that stress activates upstream hematopoietic stem cells [66]. Riddell et al. demonstrated that acute psychological stress in humans affects mobilisation of HSC cells and EPCs (endothelial PCs), as well as an increase in the number of monocytes and lymphocytes, but they did not observe an increase in circulating stem cells after administration of B-agonist receptor [67]. The participants of our study experienced chronic stress (duration of 1564 ± 2297 days), which was probably the cause of the observed differences between our results and those obtained by Riddell et al.

In the study by Kucharska-Mazur et al., they looked at selected sub-populations of stem cells and factors responsible for their movements in patients with their first psychotic episode. They observed an increase in the number of VSELs CD 34 + compared with the control group. Applied treatment did not result in a reduction in the number of these stem cells. Established deviations can be potentially treated as an indicator of a disturbance within the development process of the brain, which remains in line with Weinberger’s theory of schizophrenia as a neurodevelopmental disorder [22].

In the course of their research, Ferensztajn-Rochowiak et al. looked at the behavior of stem cells in patients with bipolar disorder. In the group of patients who did not receive lithium, the levels of VSELs CD 34 + were significantly higher compared to the control group, and they correlated with the duration of the disease [68, 69]. If we compare the results obtained in patients with panic disorder, then the study of stem cells allows to differentiate qualitatively between psychotic and anxiety disorders.

Next, we dealt with the influence of pharmacotherapy on the number of stem cells. We only observed that the level of VSELs (Lin−/CD45−/CD133 +) was significantly lower in the patient group before treatment as compared to after treatment, which may indicate the role of the administered pharmacotherapy (Table 4); from literature data we know that e.g. antidepressant treatment causes increased neurogenesis [23, 24].

**Table 4:** Comparison of stem cell numbers and factors responsible for their movement in PG BT vs PG AT

	PG BT (*n* = 30)	PG AT (*n* = 30)	P
VSEL (Lin−/CD45−/CD34+) [No.of cells/μl]	0,155 ± 0,079	0,158 ± 0,064	0,797
VSEL (Lin−/CD45/CD133+) [No.of cells/μl]	0,102 ± 0,048	0,134 ± 0,074	0,0243
HSCs (Lin−/CD45+/CD34+) [No.of cells/μl]	1173 ± 0,522	1265 ± 0,62	0,245
HSCs (Lin/CD45+/AC133+) [No.of cells/μl]	0,860 ± 0,43	0,897 ± 0,45	0,416
C3a [ng/ml]	469,56 ± 116,6	449,9 ± 86,1	0,147
C5a [ng/ml]	27 ± 13	26,98 ± 12,6	1,0
C5b-9 [ng/ml]	73,97 ± 77,29	61,43 ± 39,7	0,36
SDF-1 [pg/ml]	2101 ± 401	2057,4 ± 422	0,496
S1P [μg/ml]	2,05 ± 0,38	2,00 ± 0,32	0,465

*PG* patient group, *CG* control group, *BT* before treatment, *AT* after treatment

Factors responsible for the movement of stem cells in the body are, among others, complement proteins, chemokines and lysophospholipids. In our analysis we demonstrated significantly lower levels of C3a, C5a, C5b, S1P and SDF-1 in PG compared to CG both before and after treatment (Table 3), we did not, however, manage to show any statistically significant differences when comparing the patient group before and after treatment (Table 4). As a potential cause of reduced concentrations of these substances we could suggest the impact of chronic stress. In the available literature we did not find information on the determination of the aforementioned factors in anxiety disorders, and therefore we decided to analyze the results with relation to data from the literature on psychoses.

In their study on patients with psychosis, Kucharska et al. showed significantly lower concentrations of S1P in the patient group before and after treatment as compared with the control group, and reduced levels of C3a in the patient group before treatment as compared with the control group, which increased after treatment. They did not, however, demonstrate changes in the concentrations of the other investigated factors. The results may also be used in differentiating between psychoses and anxiety disorders [22].

In her study on the complement components in psychoses, on the basis of available literature, Mayilyan pointed out varied research results, which could be accounted for by the use of clinically different patient groups [70]. In a study comparing the concentrations of C3 in paranoid schizophrenia (MB), bipolar disease in euthymia (BD) and in healthy individuals, Santos Soria et al. demonstrated a significantly higher level of C3 in patients with schizophrenia as compared with the control group and the patients with BD in euthymia. C3 levels did not differ significantly in control group compared to BP patients in euthymia [71].

This work is the first attempt to analyze the mobilization of stem cells in panic disorder. Our results are a contribution to the further analysis of the role of regeneration system in anxiety disorders. The limitation of this study is a relatively small number of participants, which delivered interesting preliminary findings but definitively requires replication studies.

### Based on our Analyses we Have Formulated the Following Conclusions

1. Long-term panic disorder reduces the mobilization of HSCs (Lin−/CD45 +/AC133 +) from the bone marrow to the peripheral blood and a short-term use of antidepressants does not increase this mobilization.
2. The number of VSELs does not constitute a differentiating factor between the patient and control group, but the use of antidepressants causes an increase in the mobilization of VSELs (Lin−/CD45−/CD133 +) to the peripheral blood.
3. In patients with panic disorder we have observed a significantly lower concentration of the investigated factors responsible for stem cell movement, and administered pharmacotherapy has not resulted in substantial changes to that concentration.
4. The study of stem cells and factors associated with their movement might be helpful in the diagnostics of panic disorder, as well as its differentiation from psychotic disorders
